# Some Analogies of the Banach Contraction Principle in Fuzzy Modular Spaces

**DOI:** 10.1155/2013/205275

**Published:** 2013-05-20

**Authors:** Kittipong Wongkum, Parin Chaipunya, Poom Kumam

**Affiliations:** Department of Mathematics, Faculty of Science, King Mongkut's University of Technology Thonburi (KMUTT), 126 Pracha Uthit Road, Bang Mod, Thung Khru, Bangkok 10140, Thailand

## Abstract

We established some theorems under the aim of deriving variants of the Banach contraction principle, using the classes of inner contractions and outer contractions, on the structure of fuzzy modular spaces.

## 1. Introduction and Preliminaries

The concept of a modular space was introduced by Nakano [1]. Soon after, Musielak and Orlicz [2] redefined and generalized the notation of modular space. After that, Kumam et al. [3–7] studied fixed points and some properties in modular spaces. In 2007, Nourouzi [8] proposed probabilistic modular spaces based on the theory of modular spaces and some researches on the Menger's probabilistic metric spaces. A pair (*X*, *ρ*) is called a *probabilistic modular space* if *X* is a real vector space and *ρ* is a mapping from *X* into the set of all distribution functions (for *x* ∈ *X*, the distribution function *ρ*(*x*) is denoted by *ρ*_*x*_, and *ρ*_*x*_(*t*) is the value *ρ*_*x*_ at *t* ∈ ℝ) satisfying the following conditions:

 (PM1) *ρ*_*x*_(0) = 0;
 (PM2) *ρ*_*x*_(*t*) = 1 for all *t* > 0 if and only if *x* = *θ*;
 (PM3) *ρ*_−*x*_(*t*) = *ρ*_*x*_(*t*);
 (PM4) *ρ*_*αx*+*βy*_(*s* + *t*) ≥ *ρ*_*x*_(*s*)∧*ρ*_*y*_(*t*) for all *x*, *y* ∈ *X* and *α*, *β*, *s*, *t* ∈ ℝ_0_^+^, *α* + *β* = 1.

For every *x* ∈ *X*, *t* > 0 and *α* ∈ ℝ∖{0}, if

(1) $$\begin{array}{c} \rho_{\alpha x}\left(t\right)=\rho_{x}\left(\frac{t}{{\left|\alpha \right|}^{\beta }}\right), \end{array}$$

where *β* ∈ (0,1], then we say that (*X*, *ρ*) is *β*-*homogeneous.*

Recently, further studies have been made on the probabilistic modular spaces. Nourouzi [8] extended the well-known Baire's Theorem to probabilistic modular spaces by using a special condition. Nourouzi [8] investigated the continuity and boundedness of linear operators defined between probabilistic modular spaces in the probabilistic sense. After that, Shen and Chen [9] following the idea of probabilistic modular space and the definition of fuzzy metric space in the sense of George and Veeramani [10], applied fuzzy concept to the classical notions of modular and modular spaces, and in 2013, Shen and Chen [9] introduced the concept of a fuzzy modular space.

**Definition 1 (Vasuki [11])**

A *fuzzy set A* in *X* is a function with domain *X* and value in [0,1].

**Definition 2 (Arul Selvaraj and Sivakumar [12])**

A *triangular norm* is a function ∗ : [0,1]×[0,1]→[0,1] satisfying, for each *a*, *b*, *c*, *d* ∈ [0,1], the following conditions:

1. *a*∗1 = *a*;
2. *a*∗*b* ≤ *c*∗*d* whenever *a* ≤ *c*, *b* ≤ *d*;
3. *a*∗*b* = *b*∗*a* and (*a*∗*b*)∗*c* = *a*∗(*b*∗*c*).

**Definition 3 (Shen and Chen [9])**

Let *V* be a real or complex vector space with a zero *θ*, ∗ a continuous triangular norm, and *μ* a fuzzy set on the product *V* × ℝ^+^. Suppose that the following properties hold for *x*, *y* ∈ *V* and *s*, *t* > 0:

 (FM1) *μ*(*x*, *t*) > 0;
 (FM2) *μ*(*x*, *t*) = 1 for all *t* > 0 if and only if *x* = *θ*;
 (FM3) *μ*(*x*, *t*) = *μ*(−*x*, *t*);
 (FM4) *μ*(*z*, *s* + *t*) ≥ *μ*(*x*, *s*)∗*μ*(*y*, *t*) whenever *z* is the convex combination between *x* and *y*;
 (FM5) the mapping *t* ↦ *μ*(*x*, *t*) is continuous at each fixed *x* ∈ *V*.

Then, we write (*V*, *μ*, ∗) to represent the space with the pre-defined properties. In particular, we call *μ* a *fuzzy modular* and the triple (*V*, *μ*, ∗) a *fuzzy modular space*.

It is worth noting that every fuzzy modular is non-decreasing with respect to *t* > 0.

**Example 4**

Let *X* be a real or complex vector space and *ρ* be a modular on *X*. Take the *t*-norm *a*∗*b* = min⁡⁡{*a*, *b*}. For every *t* ∈ (0, *∞*), define *μ*(*x*, *t*) = *t*/(*t* + *ρ*(*x*)) for all *x* ∈ *X*. Then (*X*, *μ*, ∗) is a *F*-modular space.

**Remark 5**

Note that the above conclusion still holds even if the *t*-norm is replaced by *a*∗*b* = *a* · *b* and *a*∗*b* = max⁡⁡{*a* + *b* − 1,0}, respectively.

Shen and Chen [9] also studied the topological properties of a fuzzy modular space with a special property that for every *x* ∈ *V* and a non-zero real *λ*, the equality

(2) $$\begin{array}{c} \mu \left(\lambda x,t\right)=\mu \left(x,\frac{t}{{\left|\lambda \right|}^{\beta }}\right) \end{array}$$

holds for some fixed *β* ∈ (0,1]. If the fuzzy modular *μ* has this property, we shall say that it is *β-homogeneous*.

The *μ-ball* in (*V*, *μ*, ∗) is the set of the form

(3) $$\begin{array}{c} B\left(x,r,t\right):=\left\{y\in V;\mu \left(x-y,t\right)>1-r\right\}, \end{array}$$

where *r* ∈ (0,1) and *t* > 0. Now, suppose that *μ* is *β*-homogeneous for some *β* ∈ (0,1]. According to Shen and Chen [9], the family *𝔅* of all *μ*-balls forms a base for a first-countable Hausdorff topology, written as *𝒯*_*μ*_. With the notion of the *μ*-balls, it is easy to see that a sequence (*x*_*n*_) in *V* *μ-converges * (i.e., it converges in the topology *𝒯*_*μ*_) to its *μ-limit x* ∈ *V* if and only if *μ*(*x* − *x*_*n*_, *t*) → 1 as *n* → *∞* for all *t* > 0. Note here that the *μ*-limit is unique if it does exists after all. It is then natural to say that (*x*_*n*_) is *μ-Cauchy* if for any given *ɛ* ∈ (0,1) and *t* > 0, there exists *N* ∈ *ℕ* with *μ*(*x*_*m*_ − *x*_*n*_, *t*) > 1 − *ɛ* whenever *m*, *n* > *N*.

At this point, let us turn to a typical example of a triangular norm which is defined by (*a*∗*b*) = min⁡⁡{*a*, *b*}. This triangular norm has a very special property that if ∗′ is an arbitrary triangular norm, then (*a*∗′*b*)≤(*a*∗*b*) for all *a*, *b* ∈ [0,1]. With this property, it is suitable to call this ∗ a *strongest triangular norm*. As is claimed by Shen and Chen [9], if *V* is a real vector space equipped with a *β*-homogeneous fuzzy modular *μ* and a strongest triangular norm ∗, then a *μ*-convergent sequence is *μ*-Cauchy. The authors also mentioned that if ∗ is not the strongest one, such implementation is not always true.

The space (*V*, *μ*, ∗) is said to be *μ-complete* if *μ*-Cauchy sequences actually *μ*-converges. We note that it makes more sense if we require a complete fuzzy modular space to be equipped with the strongest triangular norm.

A mapping *f* from *V* into itself is said to be an *inner contraction* if there exists a positive constant *q* < 1 such that

(4) $$\begin{array}{c} \mu \left(fx-fy,qt\right)\geq \mu \left(x-y,t\right), \end{array}$$

for all *x*, *y* ∈ *V* and *t* > 0.

On the other hand, *f* is said to be an *outer contraction* if there exists a positive constant *q* < 1 such that

(5) $$\begin{array}{c} \mu \left(fx-fy,t\right)-1\geq q\left(\mu \left(x-y,t\right)-1\right), \end{array}$$

for all *x*, *y* ∈ *V* and *t* > 0.

In this paper, we shall be working under the aim of deriving some variants of the Banach contraction principle, by using the classes of mappings defined above, on the structure of fuzzy modular spaces.

## 2. Main Results

We divide this section into two parts, discussing independently about the two main categories of our contractions predefined in the previous section. Notice that it is clear from the definition that every fuzzy metric space is in turn a fuzzy modular space. Hence, our results are also supplied with corollaries in fuzzy metric spaces. We shall, however, omit such consequences since they are obvious.

### 2.1. Fixed-Point Theorem for an Inner Contraction

**Theorem 6**

Let *V* be a real vector space equipped with a *β*-homogeneous fuzzy modular *μ* and the strongest triangular norm ∗ such that (*V*, *μ*, ∗) is *μ*-complete. Suppose that at each *x* ∈ *V*, *μ*(*x*, *t*) → 1 as *t* → *∞*. If *f* : *V* → *V* is an inner contraction with constant *q* ∈ (0,1), then *f* has a unique fixed point.

**Proof**

Given a point *x*_0_ ∈ *V*, we suppose that *f* ^*n*^ *x*_0_ ≠ *f* ^*n*+1^ *x*_0_ for all *n* ∈ *ℕ*. Let *t* > 0, observe that

(6) μ(fnx0−fn+1x0,t)≥μ(fn−1x0−fnx0,tq)≥μ(fn−2x0−fn−1x0,tq2)⋮≥μ(x0−fx0,tqn).

As *n* → *∞*, we have *f* ^*n*^ *x*_0_ − *f* ^*n*+1^ *x*_0_ → *θ* for every *t* > 0. That is, for any given *t* > 0 and *ɛ* ∈ (0,1), there exists *N* ∈ *ℕ* such that

(7) $$\begin{array}{c} \mu \left(f^{N}x_{0}-f^{N+1}x_{0},t\right)\geq \mu \left(f^{N}x_{0}-f^{N+1}x_{0},\frac{t}{2^{\beta +1}}\right)>1-ɛ. \end{array}$$

We now claim to show by induction that *μ*(*f* ^*N*^ *x*_0_ − *f* ^*N*+*p*^ *x*_0_, *t*/2^*β*+1^) > 1 − *ɛ* for all *p* ∈ *ℕ*. Let us assume first that *μ*(*f* ^*N*^ *x*_0_ − *f* ^*N*+*j*^ *x*_0_, *t*) > 1 − *ɛ* holds at some *j* ∈ *ℕ*. Observe that

(8) μ(fNx0−fN+j+1x0,t)  =μ(12(fNx0−fN+1x0)+12(fN+1x0−fN+j+1x0),t2β)  ≥μ(fNx0−fN+1x0,t2β+1)∗μ(fN+1x0−fN+j+1x0,t2β+1)  ≥μ(fNx0−fN+1x0,t2β+1)∗μ(fNx0−fN+jx0,t2β+1·q)  ≥μ(fNx0−fN+1x0,t2β+1)∗μ(fNx0−fN+jx0,t2β+1)  >(1−ɛ)∗(1−ɛ)  =1−ɛ.

We have thus proved our claim.

Next, we shall show that (*f* ^*n*^ *x*_0_) is Cauchy. Let *t* > 0 and *ɛ* ∈ (0,1) be arbitrary, and we choose *N* ∈ *ℕ* according to the claim given above. For *n* > *m* > *N*, we may write *m* = *N* + *s* and *n* = *N* + *s* + *t*, for some *s*, *t* ∈ *ℕ*. Now, consider that

(9) μ(fmx0−fnx0,t)=μ(fN+sx0−fN+s+tx0,t)≥μ(fNx0−fN+tx0,tqs)≥μ(fNx0−fN+tx0,t)>1−ɛ.

Thus, (*f* ^*n*^ *x*_0_) is Cauchy, and so the *μ*-completeness yields that *f* ^*n*^ *x*_0_ → *x*_∗_ for some *x*_∗_ ∈ *V*. It follows that

(10) lim⁡n→∞μ(fn+1x0−fx∗,t)≥lim⁡n→∞μ(fnx0−x∗,tq)≥lim⁡n→∞μ(fnx0−x∗,t)=1,       ∀t>0.

This means *fx*_∗_ = *x*_∗_, since *𝒯*_*μ*_ is Hausdorff. To show that the fixed point of *f* is unique, assume that *y*_∗_ ∈ *V* is a fixed point of *f* as well. Finally, we obtain that

(11) μ(x∗−y∗,t)=μ(fnx∗−fny∗,t)≥μ(x∗−y∗,tqn)→1, ∀t>0.

Therefore, it must be the case that *x*_∗_ = *y*_∗_, and so the conclusion is fulfilled.

### 2.2. Fixed-Point Theorem for an Outer Contraction

For this part, we consider a weaker form of a *μ*-Cauchy sequence, namely, a *μ-G-Cauchy sequence*. This concept has been used all along together with the notion of fuzzy spaces. For a fuzzy modular space *V*, (*x*_*n*_) is called a *μ*-*G*-Cauchy sequence if for each fixed *p* ∈ *ℕ* and *t* > 0, we have lim⁡_*n*→*∞*_(*x*_*n*+*p*_, *x*_*n*_, *t*) = 1. If every *μ*-*G*-Cauchy sequence *μ*-converges, *V* is said to be *μ-G-complete*. It is to be noted that the notion of *μ*-*G*-completeness is slightly stronger than the ordinary completeness. It is enough to see that every *μ*-Cauchy sequence is also a *μ*-*G*-Cauchy sequence. For our result, it is still a question whether or not the *μ*-*G*-completeness assumption can be weakened.

**Theorem 7**

Let *V* be a real vector space equipped with a *β*-homogeneous fuzzy modular *μ* and the strongest triangular norm ∗ such that (*V*, *μ*, ∗) is *μ*-*G*-complete. If *f* : *V* → *V* is an outer contraction with constant *q* ∈ (0,1), then *f* has a unique fixed point.

**Proof**

Given a point *x*_0_ ∈ *V*, we suppose that *f* ^*n*^ *x*_0_ ≠ *f* ^*n*+1^ *x*_0_ for all *n* ∈ *ℕ*. By the definition of an outer contraction, we can rewrite this notion in the following:

(12) $$\begin{array}{c} \mu \left(fx-fy,t\right)\geq q\mu \left(x-y,t\right)+\left(1-q\right), \end{array}$$

for all *x*, *y* ∈ *V* and *t* > 0.

Let *t* > 0, observe that

(13) μ(fnx0−fn+1x0,t)≥qμ(fn−1x0−fnx0,t)+(1−q)≥q2μ(fn−2x0−fn−1x0,t) +q(1−q)+(1−q)⋮≥qnμ(x0−fx0,t)+∑k=0n−1qk(1−q).

As *n* → *∞*, we have

(14) $$\begin{array}{c} \mu \left(f^{n}x_{0}-f^{n+1}x_{0},t\right)>1-ɛ,\;\text{for}\;\;n\;\;\text{being}\;\text{sufficiently}\;\;\text{large}. \end{array}$$

Next, we shall show that (*f* ^*n*^ *x*_0_) is Cauchy. Let *t* > 0 and *ɛ* ∈ (0,1) be arbitrary, and we choose *N* ∈ *ℕ*. For *n* > *N*, *n* ∈ *ℕ* and for each *p* > 0. Now, consider that

(15) μ(fnx0−fn+px0,t)≥μ(fnx0−fn+1x0,t2β+1)∗μ(fn+1x0−fn+px0,t2β+1)≥μ(fnx0−fn+1x0,t2β+1)∗μ(fn+1x0−fn+2x0,t22(β+1)) ∗μ(fn+2x0−fn+px0,t22(β+1))⋮≥μ(fnx0−fn+1x0,t2β+1)∗μ(fn+1x0−fn+2x0,t22(β+1)) ∗μ(fn+2x0−fn+3x0,t23(β+1)) ∗⋯∗μ(fn+p−1x0−fn+px0,t2(p−1)(β+1))>(1−ɛ)∗(1−ɛ)∗⋯∗(1−ɛ)=1−ɛ.

Thus, the sequence (*f* ^*n*^ *x*_0_) is Cauchy, and so the *μ*-completeness yields that *f* ^*n*^ *x*_0_ → *x*_∗_ for some *x*_∗_ ∈ *V*. It follows that

(16) μ(fn+1x0−fx∗,t)≥qμ(fnx0−x∗,t)+(1−q),                 ∀t>0.

Taking *n* → *∞*, we have

(17) $$\begin{array}{c} \mu \left(f^{n+1}x_{0}-fx_{*},t\right)\to 1. \end{array}$$

This means *fx*_∗_ = *x*_∗_, since *𝒯*_*μ*_ is Hausdorff. To show that the fixed point of *f* is unique, assume that *y*_∗_ ∈ *V* is a fixed point of *f* as well. Finally, we obtain that

(18) μ(x∗−y∗,t)=μ(fx∗−fy∗,t)≥qμ(x∗−y∗,t)+(1−q).

Hence, we have *μ*(*x*_∗_ − *y*_∗_, *t*)(1 − *q*)≥(1 − *q*) which implies that *μ*(*x*_∗_ − *y*_∗_, *t*) = 1. Therefore, it must be the case that *x*_∗_ = *y*_∗_, and so the conclusion is fulfilled.

**Open Question 1**

Is Theorem 7 true under the assumption that *V* is *μ*-complete as in Theorem 6?
